# Mitigating impact of *Glycyrrhiza glabra* on virulent Newcastle disease virus challenge in chickens: clinical studies, histopathological alterations and molecular docking

**DOI:** 10.1007/s11259-024-10530-w

**Published:** 2024-09-24

**Authors:** Marwa I. Abdel Haleem, Mohamed M. S. Gaballa, Ali H. El-Far, Hanan A. A. Taie, Gehad E. Elshopakey

**Affiliations:** 1 Department of Avian and Rabbit Diseases, Faculty of Veterinary Medicine, Benha University, Benha, 13736 Egypt; 2 Department of Pathology, Faculty of Veterinary Medicine, Benha University, Benha, 13736 Egypt; 3 Department of Biochemistry, Faculty of Veterinary Medicine, Damanhour, University, Damanhour, 22511 Egypt; 4 Plant Biochemistry Department, National Research Centre, 33 El-Bohouth St. (Former El- Tahrir St.), Dokki, Giza, 12622 Egypt; 5 Department of Clinical Pathology, Faculty of Veterinary Medicine, Mansoura University, Mansoura, 35516 Egypt

**Keywords:** Licorice, Liver and kidney functions, Viral shedding, Oxidative stress, Histopathology, Molecular docking

## Abstract

**Background:**

Newcastle disease (ND) is widely regarded as one of the most virulent and destructive viral infections that create chaos in the poultry industry and cause widespread epidemics and consequentially debilitating economic losses on a global scale in terms of chicken products. The current experiment evaluates the protective effect of *Glycyrrhiza glabra* ( *G. glabra*) against the Newcastle disease virus (NDV) in chickens. Ninety (90) 1-day-old SPF chicks were treated according to ethical approval (BUFVTM 05-02-22) as follows (1) non-treated non-challenged control group; (2) NDV group: Challenged with genotype VII ND virus; and (3) LE/NDV group: Challenged with the virus and intermittently treated with powdered extract of *G. glabra* roots (LE) in drinking water (0.5 g/L) before and after viral challenge.

**Result:**

The water medication of NDV-challenged chicks has resulted in a significant decrease in the severity of clinical symptoms, morbidity, and mortality rates, as well as the quantity of virus shed, compared with the NDV group. Treatment with LE has led to a significant reduction in serum ALT and AST activities, blood glucose level, urea, and creatinine, and significant restoration of serum proteins. In addition, the treatment has resulted in a decrease in MDA and NO levels, as well as an increase in T-SOD and catalase activities compared with untreated challenged chicks. LE decreased IFN-γ and TLR-3 gene expression in comparison with the NDV group. The treated challenged birds had fewer macroscopically detectable lesions in their respiratory, digestive, and lymphoid organs than the untreated challenged birds. Microscopically, the LE/NDV group exhibited mild to moderate pathological changes in the respiratory and digestive systems as well as lymphoid tissues, in contrast to the NDV group, which exhibited severe pathological changes. Furthermore, molecular docking assessment proved the efficacy of *G. glabra* against viral proliferation and invasion.

**Conclusion:**

We concluded that *Glycyrrhiza glabra* powdered extract at a dose of 0.5 g/L drinking water can effectively mitigate the debilitating effects of Newcastle disease in chickens.

## Introduction

Newcastle disease is an infectious as well as highly contagious disease that threatens poultry farms worldwide (Rasoli et al. 2014). Many birds are infected, but chickens are the most vulnerable hosts (Okoroafor et al. 2018). It is critical to the international economic and social situation as well as a threat to human health, prompting the World Organization for Animal Health (OIE) to include it on the list of notifiable diseases OIE (2005). NDV was recorded in Egypt for the first time in 1947 (Daubney and Mansy 1948), and many outbreaks followed. The incriminated cause of ND is a single-stranded RNA-enveloped virus from the genus *Orthoavulavirus*, subfamily *Avulavirinae* and family *Paramyxoviridae* (Swayne et al. 2020). According to the clinical picture in chickens, NDV strains have variable fluctuating virulence that ranges from low to moderate to high (Alexander 1998). The NDV primarily infects the respiratory and digestive tracts, which leads to several clinical manifestations such as increased mortality (up to 100%) and morbidity, a dramatic drop in performance parameters (weight gain and feed conversion), loss in reproductive performance, as well as dyspnea, coughing, and greenish diarrhea (Liu et al. 2012, 2019a).

The most effective strategies for managing NDV are vaccination (oral, ocular, and intranasal) and biosecurity (Eze et al. 2014). However, ND epidemics are still happening in many countries, even in vaccinated farms, due to short-lived humoral antibodies (Guo et al. 2021) as well as a loss of vaccine efficiency once strains eventually evolve (Lv et al. 2019).

Licorice (*Glycyrrhiza glabra*) is a Greek term that translates as “sweet root” The herb has a long history of being utilized by numerous nations all over the globe, including the Middle East, where it was first documented in Arabic medicine as a treatment for various human diseases as early as the Middle Ages (Fiore et al. 2008; Wang et al. 2015). More than 20 triterpenes, 300 flavonoids, and 73 bioactive compounds are extracted from *G. glabra* (Li et al. 2011; Liu et al. 2013; Yang et al. 2016). Many of these substances are believed to possess antiviral, antibacterial, antioxidant, immunomodulatory, hepatoprotective, and anticarcinogenic properties (Hao et al. 2020; Mutaillifu et al. 2020; Pan et al. 2020; Pastorino et al. 2018). The *G. glabra* is known to possess antiviral activity against duck hepatitis virus (Okda et al. 2013; Soufy et al. 2012), infectious bronchitis virus, as well as infectious bursal disease virus (Li et al. 2009). Further to that, the immunomodulatory effect of *G. glabra* extract against the NDV vaccine in chickens was previously recorded (Wu et al. 2022). The proven antiviral efficacy of some components found in *G. glabra* is linked to their ability to decrease the fluidity of viral membranes, generate interferon-γ (IFN-γ), hinder phosphorylating enzymes, and reduce the period of viral latency (Fiore et al. 2008; Harada 2005; Lin et al. 2009).

With this historical backdrop in mind, the current research aimed to investigate the immunomodulatory and protective effect of *G. glabra* powdered root extract against the challenge with one of the currently circulating Egyptian velogenic strain of NDV in specific pathogen free (SPF) chicks.

## Materials and methods

### Ethical approval

The animal welfare committee at Benha University’s Faculty of Veterinary Medicine established the guidelines for this study’s experimental procedures and approved it with the number of BUFVTM 05-02-22.

### Phytochemical analysis of LE

The powdered extract of *G. glabra* roots was purchased from Al-Abji Factory, Cairo, Egypt, for the extraction of vegetables and essential oils. The extract was evaluated inside The National Research Center’s laboratories in Giza, Egypt.

The Folin-Ciocalteu procedure was used to determine the total phenol (TP) content and results were expressed in milligrams of gallic acid equivalent (GAE) per gram of dry extract weight (Taha et al. 2015).Total flavonoids were calculated (Ordonez et al. 2006). The total saponin content was measured calorimetrically (Makkar et al. 2007). The results were given in mg diosgenin equivalent per gram of sample (mg DE/g). With minor modifications, total terpenoids were estimated and results were expressed in milligrams of linalool (LE) per gram of dry sample (Koleva et al. 2002).

### Virus strain and experimental challenge

A previously isolated and characterized virulent genotype VII ND virus (NDV/CH/EG-Q/11/2018) belonging to class II with accession number MN137991 was used to challenge experimental groups (Desouky et al. 2020). The virus was propagated (10^6.5^ EID50/1 ml) according to previously described method (OIE 2012). The experimental chicks were challenged with 10^6.5^ EID50/1 ml via the oculonasal route at the age of 33 days (Rasoli et al. 2014). Meanwhile, the chicks in the negative control group were administered 1 ml of phosphate-buffered saline using the same route.

### Experimental chicks and design

Ninety (90) 1-day-old White Leghorn layer SPF chicks were purchased from the Agricultural Research Center in Kom Oshim, Fayoum, Egypt. The birds were kept under hygienic, disinfected conditions in the Animal Research Center at Benha University’s Faculty of Veterinary Medicine in Qalyubia, Egypt. Birds were raised on wood shaving-bedded floor pens (7–10 birds /1 m^2^), artificial lighting was used for 23 h daily during the trial. For the first week, brooding temperature was 35–32 °C using heaters; then it was gradually lowered weekly until it reached 24 °C till the experiment ended. The relative humidity was adjusted to a range of 65–75%. Windows and low-pressure fans were used to ventilate rooms. Each compartment had enough feeders and waterers. An ad libitum approach was employed to deliver fresh feed and water. The health condition of all the chicks was closely monitored by performing daily health checks and all efforts were made to minimize suffering.

The chicks were randomly allocated into three groups of 30 chicks per group in 5 replicates (6 birds per replicate) and treated as follows: (1) non-treated non-challenged control group; (2) NDV group: Challenged with NDV at 33 day old; and (3) LE/NDV group: Challenged with NDV at 33 day old and intermittently treated with LE in drinking water (0.5 g/L) 3 days per week for 4 weeks before challenge and for 5 days after challenge (33–37 d of age). The control group, which was not subjected to any treatment or challenges, was maintained in isolation from the experimental area from day 0 of experiment. To mitigate artificial errors and enhance experimental replicability, we choose to establish a sample size of five birds for each test index (Swayne et al. 2004; Olesen et al. 2018).

### Clinical examination (clinical signs, mortality rate, and lesion scoring)

The birds were observed twice daily by trained professional for 7 days post challenge (dpc) to record the onset and the severity of clinical signs such as depression, dropped wings, reluctance to move, digestive disturbance signs (greenish diarrhea), and nervous signs. The mortalities were recorded daily for 7 days after the challenge. The total mortality rate was determined by keeping track of the number of dead birds relative to the total number of birds in the group for 7 dpc and multiplying that number by 100.

At 5th and 7th dpc, a total of five birds were chosen at random from each group and thereafter subjected to euthanasia via neck dislocation. Breast muscles, trachea, lungs, proventriculus, intestine, and cecal tonsil (CT) (*n =* 5) were assessed for lesions (Afonso et al. 2012). A score of 0 indicated no lesions, 1 indicated mild inflammation, 2 indicated moderate inflammation, and 3 indicated severe inflammation in the breast muscles, trachea, and lungs, respectively. The proventriculus score ranged from 0 (normal) to 5 (severe bleeding), with 1 representing inflammation and 2 representing edema. Intestinal health was rated on a scale from 0 (normal) to 3 (many ulcers). Finally, CTs were given a score between 0 (normal) and 3 (severe hemorrhage with obvious ulcers) based on severity.

### Virus excretion

Using the RNeasy^®^ Mini Kit (Qiagen) for reverse transcription-Real time polymerase chain reaction (RRT-qPCR) (Wise et al. 2004), five oropharyngeal swabs were obtained from each group at 3, 5, and 7 dpc to estimate virus shedding (Nahed et al. 2020).

### Blood samples collection

Five birds were sampled from each group at 5 dpc by drawing blood from their jugular veins. In order to count the number of red blood cells, half of each sample was drawn into a tube pretreated with dipotassium EDTA for blood cell count. The serum was centrifuged out of the other half and stored at -80 °C to preserve its biochemical and immunological properties.

### Blood cell count

Erythrocytes (RBCs), leukocytes (WBCs), and differential leukocytic counts, as well as packed cell volume (PCV), hemoglobin (Hb), and platelets count were determined according to Lamb (1991).

### Liver and kidney markers

Serum levels of total protein (Catalog No.; MBS165636), albumin (Catalog No.; MBS263399), and glucose (Catalog No.; MBS7204522) were estimated spectrophotometrically (Lambda EZ201; Perkin Elmer) using corresponding kits obtained from the MyBioSource company (California, USA) and following the standard protocol of their respective pamphlets. In addition, the levels of creatinine and uric acid were assessed (Bartles and Bohmer 1972; Prætorius and Poulsen 1953).

### Oxidative stress/antioxidant parameters

At 5th dpc, parts of the trachea, lung, proventriculus, and intestine tissues were collected, washed three times using cold NaCl solution (0.9%), and homogenized in cold phosphate buffer saline (PBS) (PH 7.5). Later, the homogenates were cold centrifuged for about 15 min at 3000 r.p.m and the supernatants were carefully collected in a clean tube to be used in the evaluation of antioxidants and oxidative stress parameters (Fernandez-Botran et al. 2002). The homogenate protein level was determined using the protocols described by Bradford (1976). The levels of malondialdehyde (MDA, catalog No.; MD 25 29), catalase (catalog No.; CA 25 17), and superoxide dismutase (SOD, catalog No.; SD 25 21) estimated spectrophotometrically, in the tissues from trachea, lung, proventriculus, and intestine, as well as, serum concentration of nitric oxide (NO, catalog No.; NO 25 33) (*n =* 5), following the illustrated approaches of Bio-diagnostic kits (Cairo, Egypt). Using Bradford methods, the quantity of protein in the tissue homogenate was quantified (Bradford 1976).

### Serum lysozyme activity

Serum lysozyme activity was measured (Ghareghanipoora et al. 2014), based on the lysate of *Micrococcus lysodeikticus* (Sigma Co., USA), with modifications. At 25^o^C for 5 min, a mixture of serum and *M. lysodeikticus* suspension (0.2 mg/mL in 0.05 M PBS, pH 6.2) reacted. The optical density was then measured every minute for five minutes at 540 nm using a BM 5010 photometer. Using a calibration curve constructed using various dilutions of lyophilized chicken egg-white lysozyme (Sigma Co., USA), the serum lysozyme concentration was determined.

### DNA extraction and reverse transcription polymerase chain reaction (RT-PCR) assessment

Using RT-qPCR, the expression of toll-like receptor 3 (TLR-3) and IFN-γ messenger RNAs in the trachea, lung, intestine, and proventriculus was determined. To normalize the data, glyceraldehyde-3-phosphate dehydrogenase (GAPDH) was used as the reference housekeeping gene. Following the manufacturer’s instructions, RNA was extracted from various tissues using TRIzol reagent (Invitrogen Life Technologies) and reverse-transcribed into cDNA using TOPscriptTM RT DryMIX (Enzynomics co Ltd, Korea). To determine gene expression, Quanti Fast SYBR Green RT-PCR reagent (Qiagen) was utilized for qRT-PCR. Eurofins Genomics (Eurofins Genomics, Germany) designed the sense and antisense primers in Table 1. qRT-PCR analyses were conducted in triplicate using the ViiATM 7 System (Thermo Fisher Scientific). Using the Ct method, the fold-change was estimated (Livak and Schmittgen 2001).

**Table 1:** The PCR primers sequences of the studied genes

Gene	Accession number	Sense (5`-3`)	Antisense (5`-3`)
*IFN-γ*	Y079221	GTAAGGAACTTCAGCCATTG	GACGAATGAACTTCATCTGCC
*TLR-3*	NM001011691	CCGCCTAAATATCACGGTAC	GCGTCATAATCAAACACTCCC
*GAPDH*	NM204305	ATACACAGAGGACCAGGTTG	AAACTCATTGTCATACCAGG

### Histopathological examination

Trachea, lungs, proventriculus, intestinal tract (mid small intestine), and cecal tonsil, 5 per group from each organ, were collected from euthanized birds at 5 and 7 dpc. These samples were fixed with 4% paraformaldehyde then processed and stained with hematoxylin and eosin stain (H & E) (Bancroft et al. 1994).

### Molecular docking assessment

The three-dimensional structures of NDV’s hemagglutinin-neuraminidase, fusion glycoprotein F0, and RNA-directed RNA polymerase L and *Gallus gallus*’s TLR-3 were retrieved from RCSB Protein Data Bank (https://www.rcsb.org/) and AlphaFold (https://alphafold.ebi.ac.uk/) protein structure databases. Proteins were prepared for docking using MOE 2015.10 (Vilar et al. 2008) software. In addition, the three-dimensional structures of *G. glabra* bioactive compounds were retrieved from LOTUS: Natural Products Online (https://lotus.naturalproducts.net/) database. Furthermore, the molecular docking and protein-ligands interactions were done using MOE software.

### Statistical analyses

A one-way ANOVA and *Tukey’s post hoc* were performed. Post Hoc tests were used to investigate differences between groups in virus shedding and clinical parameters. For the analysis, the statistical software application SPSS for Windows (version 20.0; SPSS Inc., Chicago, IL, USA) was utilized. *p* < 0.05 was specified as the threshold for statistical significance between mean values. The values were expressed as mean ± standard error.

## Results

### Extract analysis

The *G. glabra* root extract analysis (Table 2) confirmed the presence of many secondary metabolites, with total phenols (47.320.27 mg gallic /g) being the main constituent, while total flavonoids and tannins were found to be 15.440.24 mg Querstine /g and 8.830.10 mg gallic /g, respectively. Total saponin in *G. glabra* extract was found to be 239.641.88 mg diosgenin/g, with total terpenoids in the investigated extract being 11.740.25 mg linalool/g.

**Table 2:** Total content of active compounds in the *G. glabra* extract

Total phenols (mg gallic /g D.W.)	Total flavonoids mg Quercetin /g D.W.	Total tannins (mg gallic acid/g D.W.)	Total saponin (mg diosgenin /g D.W.)	Total terpenoids (mg linalool /g D.W.)
47.32 ± 0.27	15.44 ± 0.24	8.83 ± 0.10	239.64 ± 1.88	11.74 ± 0.25

Values are given as mean (*n =* 5) ± SD

### Clinical signs and mortality percent

Clinical symptoms began to appear in challenged birds of the NDV group in form of green diarrhea on the third day of challenge. The challenged groups (NDV and LE/NDV) displayed general disease signs (depression, off food, closed eyes, reluctance to move, ruffled feathers, and dropped wings) on the fourth day, as well as green diarrhea and neurological problems represented by lateral recumbency and inability to stand or moving. The NDV group had a higher morbidity rate than the LE/NDV group. The symptoms persisted until the seventh day of infection and complete paralysis of the legs and/or head in the NDV group on the fifth day was recorded, with some cases of head tilting appearing as early as the sixth day in the LE/NDV group. The negative control group’s birds were all clinically normal. Mortalities were first observed on the 5th dpc in both challenged groups, and they persisted until the end of the monitoring period. The mortality rates at 5th and 7th dpc were recorded (Table 3). The total number of deceased chicks in the NDV group at 7 dpc was 22 of 30 birds, a 73% increase over the control group, which experienced no mortalities during the observation period. The number of deceased chicks in the LE/NDV group (12 of 30 birds, 40%) was significantly (*>p < 0.05*) lower than in the NDV group (Table 3).

**Table 3:** Effect of licorice extract supplementation on lesion score and mortality rates of SPF chicks challenged with Newcastle Disease virus

Time/Organs		Experimental groups#		
		Control	NDV	LE/NDV
5th dpc	**Breast**	0.00 ± 0.00 ^b^	3.40 ± 0.75 ^a^	1.40 ± 0.93 ^ab^
	**Trachea**	0.00 ± 0.00 ^b^	1.40 ± 0.40 ^a^	1.20 ± 0.20 ^a^
	**Lung**	0.00 ± 0.00 ^b^	1.40 ± 0.40 ^a^	0.40 ± 0.40 ^ab^
	**Proventriculus**	0.00 ± 0.00 ^b^	2.40 ± 0.51 ^a^	1.40 ± 0.25 ^a^
	**Intestine.**	0.00 ± 0.00 ^b^	2.00 ± 0.32 ^a^	1.60 ± 0.25 ^a^
	**CT**	0.00 ± 0.00 ^c^	2.80 ± 0.20 ^a^	1.60 ± 0.40 ^b^
7th dpc	**Breast**	0.00 ± 0.00 ^b^	0.80 ± 0.37 ^a^	0.00 ± 0.00^b^
	**Trachea**	0.00 ± 0.00 ^b^	1.60 ± 0.25 ^a^	0.60 ± 0.25 ^b^
	**Lung**	0.00 ± 0.00 ^a^	0.60 ± 0.40 ^a^	0.40 ± 0.25 ^a^
	**Proventriculus**	0.00 ± 0.00 ^b^	2.20 ± 0.06 ^a^	0.80 ± 0.49 ^b^
	**Intestine**	0.00 ± 0.00 ^b^	1.20 ± 0.20 ^a^	0.00 ± 0.00 ^b^
	**CT**	0.00 ± 0.00 ^b^	0.80 ± 0.37 ^a^	0.20 ± 0.20 ^ab^
Mortality	5th dpc	0	19	6
	7th dpc	0	3	6
	Total	0/30 (0%) 0.00 ± 0.00 ^c^	22/30 (73%) 7.33 ± 1.45 ^a^	12/30 (40%) 4.00 ± 0.58 ^b^

Tukey^’^s represents least significant differences between different groups at probability *p* < 0.05. Means with different superscripts (a, b, c, and d) within a row are significantly different at *p* < 0.05. Values are given as mean (*n* = 5) ± SE

#: Control group: non-treated non-challenged; NDV group: challenged with NDV; and LE/NDV group: challenged with NDV and intermittently treated with LE in drinking water (0.5 g/L) 3 days/week for 4 weeks before challenge and 5 days after challenge. dpc: day post challenge

### Lesion scoring

**Fig. 1:**
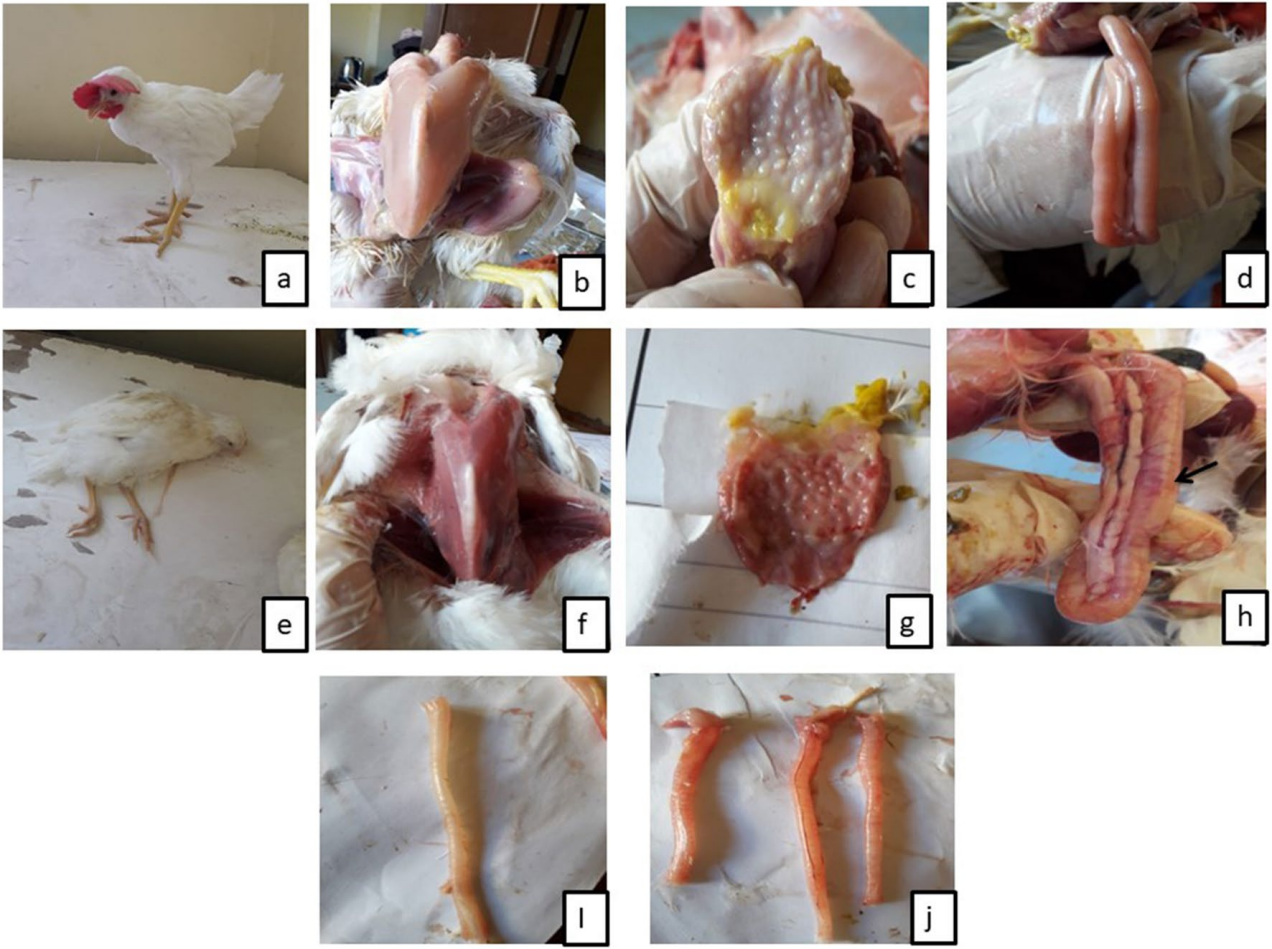
Clinical signs and lesions recorded in control and NDV challenged groups

At the 5th and 7th dpc, remarkable lesions were observed on various sections of both the respiratory and digestive systems in euthanatized birds of NDV group, in contrast to the control group, which did not exhibit any macroscopical lesions (Table 3; Fig. 1). Cecal tonsil scores were drastically (*p  < 0.05*) lower in the LE/NDV group compared with the NDV group at 5th dpc. Lesions on the breast muscle, trachea, proventriculus, and intestinal tract were remarkably (*p < 0.05*) lower in the score at 7th dpc when compared with the NDV group.

### Viral shedding

The nasopharyngeal swabs from the negative control group showed no virus shedding by RT-PCR (Table 4). The NDV group began shedding on the 3rd dpc and continued through the 5th and 7th dpc. Titers of the shedded virus on the 3rd, 5th, and 7th dpc were drastically (*p < 0.05*) lower in the LE/NDV group than in the NDV group.

**Table 4:** Effect of licorice extract supplementation on viral shedding of challenged chicks with Newcastle Disease virus

Group#/ shedding time	3 dpc	5 dpc	7 dpc
Control	0.00 ± 0.00 ^c^	0.00 ± 0.00 ^c^	0.00 ± 0.00 ^c^
NDV	32.29 ± 0.88 ^a^	29.69 ± 0.88 ^a^	28.95 ± 0.88 ^a^
LE/NDV	26.50 ± 0.88 ^b*^	26.49 ± 0.88 ^b^	24.31 ± 0.88 ^b^

Tukey^’^s represents least significant differences between different groups at probability *p* < 0.05. Means with different superscripts (a, b, c, d) within a column are significantly different at *p* < 0.05. Values are given as mean (*n = 5*) ± SE

#: Control group: non-treated non-challenged; NDV group: challenged with NDV; and LE/NDV group: challenged with NDV and intermittently treated with LE in drinking water (0.5 g/L) 3 days/week for 4 weeks before challenge and 5 days after challenge. dpc: day post challenge

### Blood cell count

A disturbance in blood cell count was recorded in NDV group as presented in the Table 5. The obtained results showed significantly lower RBCs count (*p* < 0.001), Hb (*p* < 0.001) and PCV (*p* < 0.001) values with increased count of WBCs (*p* < 0.001), lymphocyte (*p* < 0.01), heterophil (*p* < 0.001) and eosinophil (*p* < 0.01) in NDV group compared with the control one. Significant increase in MCV (*p* < 0.05) with lower MCHC in NDV-challenged birds showing features of macrocytic hypochromic anemia. All blood indices significantly returned partially toward the normal values in LE treated birds unlike the NDV group (*p* < 0.05).

**Table 5:** Effect of licorice extract supplementation on blood cell count of SPF chicks challenged with Newcastle Disease virus

Parameters	Experimental groups#		
	Control	NDV	LE/NDV
RBCs (10^6^/µL)	3.49 ± 0.12	1.62 ± 0.17 ***	2.33 ± 0.09 **
Hb (g/dl)	11.15 ± 0.35	6.89 ± 0.19 ***	7.99 ± 0.28 *
PCV (%)	38.50 ± 0.64	24.25 ± 0.93 ***	30.51 ± 0.65 **
MCV (fl.)	110.70 ± 3.42	155.99 ± 11.48 *	131.4 ± 9.30
MCHC (%)	28.89 ± 2.69	24.33 ± 2.92 *	26.21 ± 2.94
TLC (10^3^/µL)	8.88 ± 0.49	4.09 ± 0.17 ***	6.23 ± 0.09 **
Lymphocyte (10^3^/µL)	4.66 ± 0.38	2.35 ± 0.10 **	3.16 ± 0.12 **
Heterophil (10^3^/µL)	3.49 ± 0.13	1.17 ± 0.07 **	2.33 ± 0.11 *
Monocyte (10^3^/µL)	0.39 ± 0.08	0.37 ± 0.09	0.54 ± 0.13
Eosinophil (10^3^/µL)	0.26 ± 0.02	0.13 ± 0.01 **	0.15 ± 0.02 **
Basophil (10^3^/µL)	0.08 ± 0.01	0.06 ± 0.01	0.06 ± 0.02

Data were represented as Mean ± SME

NS; Non-significant, *; *p* < 0.05, **; *p* < 0.01, ***; *p* < 0.001

#: Control group: non-treated non-challenged; NDV group: Challenged with NDV; and LE/NDV group: Challenged with NDV and intermittently treated with LE in drinking water (0.5 g/L) 3 days/week for 4 weeks before challenge and 5 days after challenge

### Serum biochemical parameters

The impacts of LE supplementation on serum biochemical parameters of NDV-challenged chicks were demonstrated in the Table 6. Chicks in NDV group showed a significant increase in ALT (*p* < 0.05), AST (*p* < 0.001), glucose (*p* < 0.001), uric acid (*p* < 0.001), and creatinine (*p* < 0.001), beside lower serum total proteins (*p* < 0.01), albumin (*p* < 0.05), and globulins (*p* < 0.05) compared with control one. Interestingly, supplementation of LE significantly (*p* < 0.05) restored all biochemical parameters relative to that of the NDV group, but still higher than the control one.

**Table 6:** Effect of licorice extract supplementation on serum indices of SPF chicks challenged with Newcastle Disease virus

Parameters	Experimental groups^#^		
	Control	NDV	LE/NDV
ALT (U/L)	12.59 ± 1.22	16.13 ± 0.53 *	16.21 ± 1.17
AST (U/L)	50.57 ± 2.74	89.44 ± 2.72 ***	71.93 ± 1.51 ***
Albumin (g/dL)	2.75 ± 0.16	1.6 ± 0.14 *	2.25 ± 0.08
T. protein (g/dL)	5.17 ± 0.29	2.97 ± 0.17 **	3.58 ± 0.16 *
Globulin (g/dL)	2.42 ± 0.15	1.38 ± 0.14 *	1.34 ± 0.21 *
Glucose (mg/mL)	69.90 ± 2.65	124.27 ± 5.66 ***	100.03 ± 1.42 ***
Uric acid (mg/dL)	2.51 ± 0.17	6.04 ± 0.49 ***	3.57 ± 0.21 *
Creatinine (mg/dL)	0.61 ± 0.05	1.35 ± 0.07 ***	0.96 ± 0.04 *

Data were represented as Mean ± SME. NS; Non-significant, *; *p* < 0.05, **; *p* < 0.01, ***; *p* < 0.001

ALT, alanine aminotransferase; AST, Aspartate aminotransferase

#: Control group: non-treated non-challenged; NDV group: Challenged with NDV; and LE/NDV group: Challenged with NDV and intermittently treated with LE in drinking water (0.5 g/L) 3d /week for 4 weeks before challenge and 5 days after challenge

### Serum NO level and lysozyme activity

**Fig. 2:**
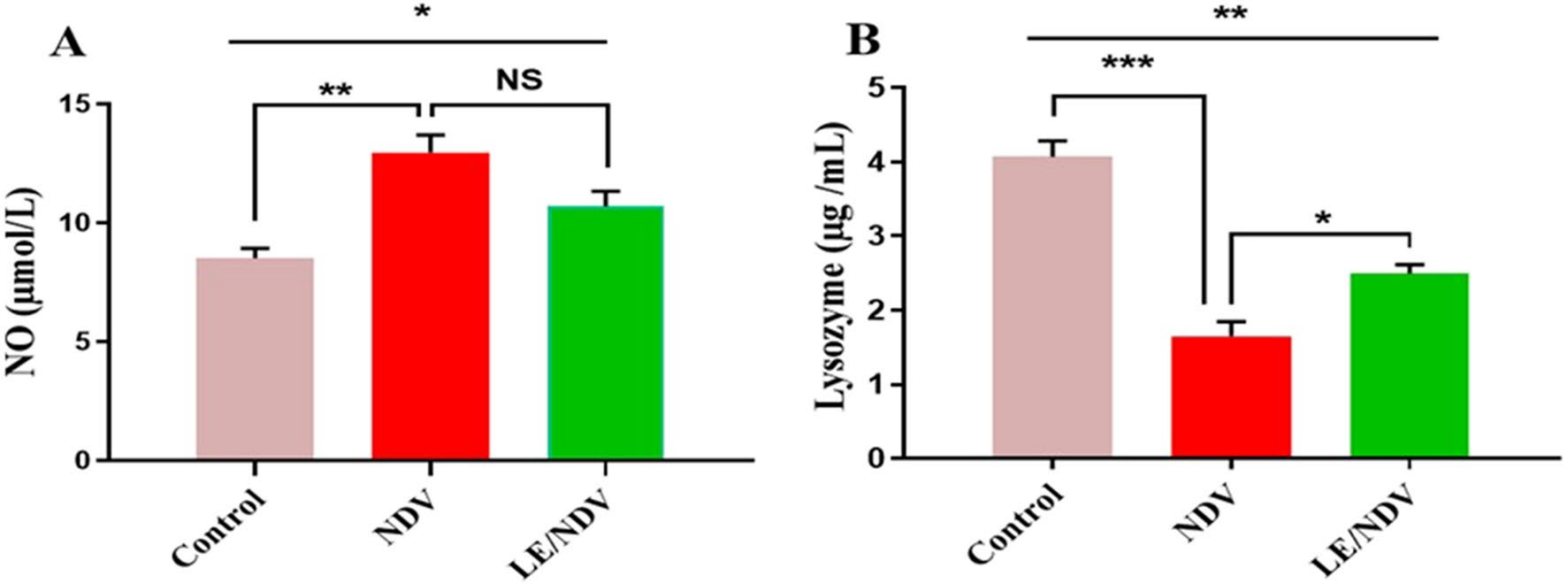
Effect of licorice extract supplementation on serum nitric oxide level (A) and lysozyme activity (B) in NDV challenged chickens. Data were analyzed using Tukey’s post hoc test (NS; Non-significant, *; *p* < 0.05, **; *p* < 0.01, ***; *p* < 0.001), and expressed as mean ± SME (*n* = 5/ group). #: Control group: Non-treated non-challenged; NDV group: Challenged with NDV; and LE/NDV group: Challenged with NDV and intermittently treated with LE in drinking water (0.5 g/L) 3d /week for 4 weeks before challenge and 5 days after challenge

A significant elevation of serum NO (*p* < 0.01) levels associated with lower lysozyme activity (*p* < 0.001) was recorded in NDV group compared with the control group (Fig. 2). The lysozyme (*p* < 0.05) activity in LE/NDV group was markedly increased compared with the NDV group (Fig. 2B).

### Oxidative/Antioxidant status

**Fig. 3:**
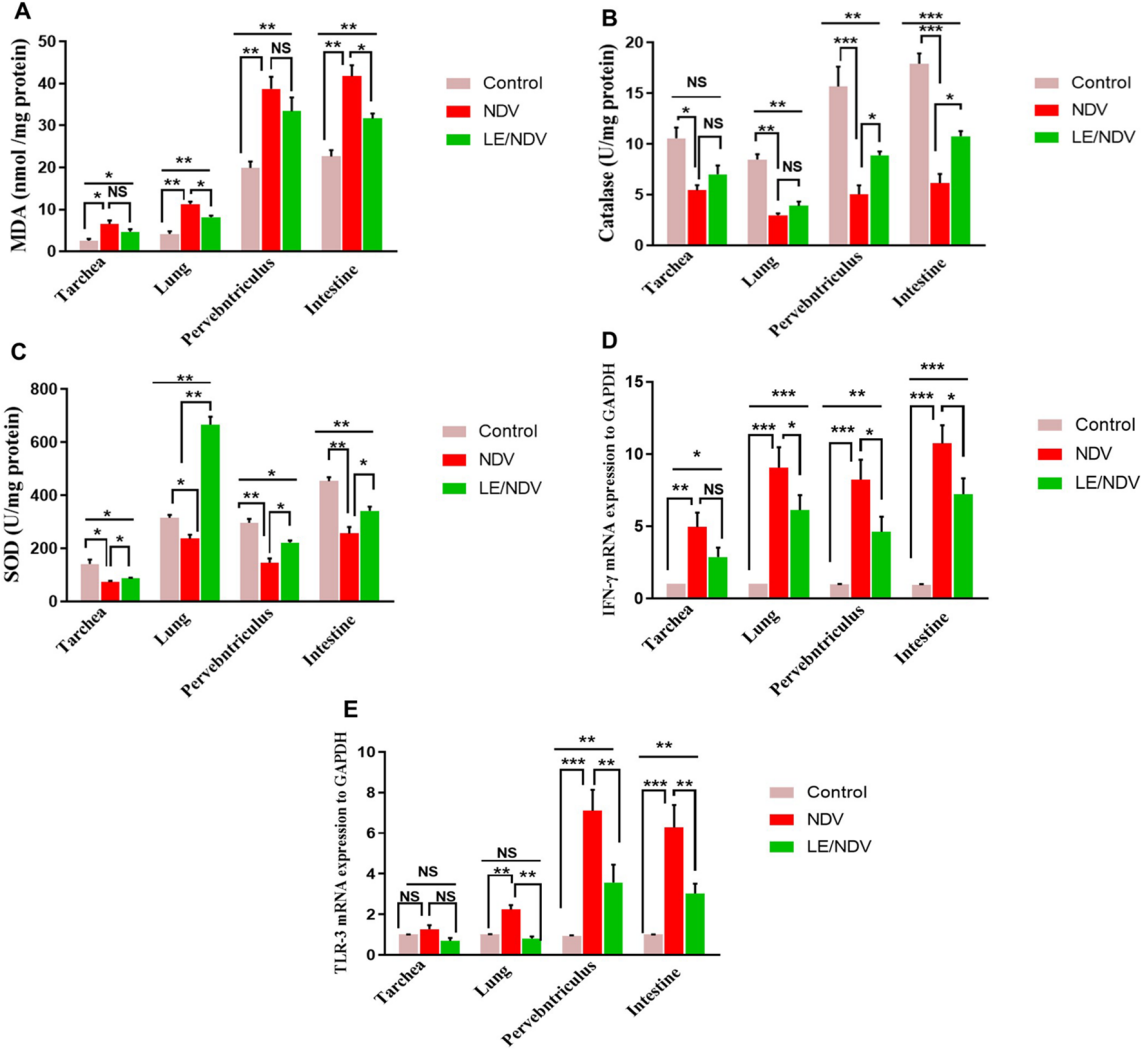
Effect of licorice extract supplementation on malondialdehyde (MDA, A), catalase (B) superoxide dismutase (SOD, C), IFN-γ (D) and TLR-3 (E) in trachea, lung, proventriculus and intestine of NDV challenged chickens. Data were analyzed using Tukey’s post hoc test (NS; Non-significant, *; *p* < 0.05, **; *p* < 0.01, ***; *p* < 0.001), and expressed as mean ± SME (*n* = 5/ group). #: Control group: Non-treated non-challenged; 2) NDV group: Challenged with NDV; and 3) LE/NDV group: Challenged with NDV and intermittently treated with LE in drinking water (0.5 g/L) 3d /week for 4 weeks before challenge and 5 days after challenge

As compared with the control, tissues from the trachea (*p* < 0.05), lungs (*p* < 0.01), proventriculus (*p* < 0.01), and intestine (*p* < 0.01) had significantly higher levels of MDA generation after NDV infection (Fig. 3A). However, in the NDV group, catalase (*p* < 0.05, *p* < 0.01, *p* < 0.001) and SOD (*p* < 0.05, *p* < 0.01) activation was considerably attenuated across all tissues compared with the control group (Fig. 3B, C). There was a significant reduction in MDA production in the lungs and the intestines of LE treated chicks (*p* < 0.05) and an increase in SOD activity in all tissues (*p* < 0.05, *p* < 0.01), but a rise in catalase activity (Fig. 3A, C) only in the proventriculus and the intestine (*p* < 0.01) was recorded.

### Differential expression of IFN-γ and TLR-3 genes

IFN-γ mRNA was up regulated in the trachea (*p* < 0.01), lung (*p* < 0.001), proventriculus (*p* < 0.001), and intestine (*p* < 0.001) of NDV group, as was TLR-3 mRNA in the lung (*p* < 0.01), proventriculus (*p* < 0.001), and intestine (*p* < 0.001). In contrast, the lung, proventriculus, and intestinal of LE/NDV birds were downregulated (Fig. 3D & E) in comparison with the NDV group (*p* < 0.05, *p* < 0.01).

### Histopathological examination

#### Trachea

**Fig. 4:**
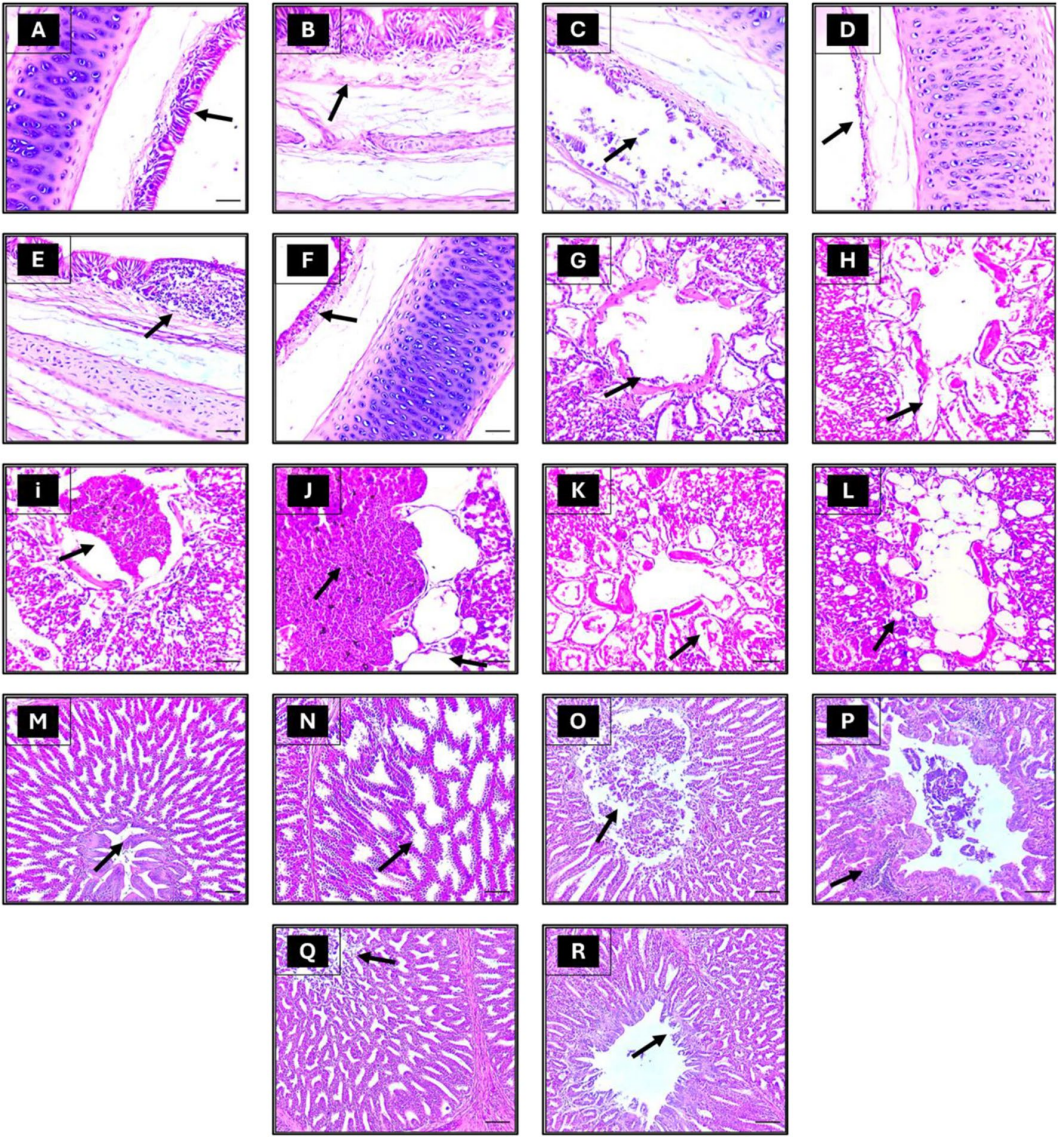
Depicts histological micrographs of different organs from chickens of distinct experimental groups (H&E [magnification, ×200]). Group 1 corresponds to the negative-control group, with specimens collected at 5- and 7-dpc (dpc) displaying typical trachea tissue with ciliated columnar epithelium (A), goblet cells, and a dense fibrous submucosa (B). Group 2 represents the positive-control group, with samples obtained at 5 and 7 dpc after being challenged solely with Newcastle disease virus (NDV) displaying severe epithelium deciliation, and desquamation (C), complete necrosis as well as edema in the lamina propria/submucosa layers (D). Finally, group 3 is the experimental group, which received licorice in addition to NDV, and samples were collected at 5 and 7 dpc with mild hyperplasia of the epithelium, lamina propria occasionally invaded with lymphocytes and an occasional germinal center (B), mild epithelial deciliation, degeneration and rounding up (F). Group 1 lung displays normal para bronchi and patent air capillaries (G, H). Group 2 lung displaying hemorrhage and inflammatory cells (i). Group 2 (positive-control) at 5 and 7 dpc, after being challenged solely with (NDV) displayed extensive bleeding into the parabronchial lumen along with collapsed and ruptured alveoli (J). Finally, group 3 is (NDV licorice). Samples were collected at 5 and 7 dpc with mild congestion and inflammatory cell infiltrations (K, L). Group 1 (Negative-control) group, with specimens collected at 5- and 7-dpc displaying the normal structure of proventriculus epithelium and glands (M, N). Group 2 represents the positive-control group, with samples obtained at 5 and 7 dpc after being challenged solely with (NDV) displaying severe necrosis and desquamation of proventriculus glands and epithelium (O) along with congestion and infiltration of inflammatory cells into the submucosa (P). Finally, group 3 is (NDV licorice), and samples were collected at 5 and 7 dpc with the improvisation of the degenerative changes in the histological structure of the proventriculus, only mild desquamation (Q, R)

The tracheal tissue sections obtained from the negative control group exhibited no noticeable alterations at both 5 and 7 dpc (Fig. 4A and B, respectively). By the 5th dpc severe lesions, primarily localized (++) in certain areas of the tissue, were evident in the trachea of NDV birds (Table 7). These lesions included inflammation, desquamation, deciliation, and epithelial hyperplasia (Fig. 4C). Even though the tracheal lesions reduced by 7th dpc, they became more distributed throughout the tissue (+++, Table 7), displaying edema in the lamina propria and submucosa layers, along with necrosis and desquamation of the tracheal mucosa (Fig. 4D).

**Table 7:** The severity of histopathological lesions in trachea, lung, proventriculus, intestine, and lymphoid follicle of the experimental chicken groups

Trachea	Lung	Proventriculus	Intestine	lymphoid follicle	Organs Chicken groups
−	−	−	−	−	G1 5 dpc
−	−	−	−	+	G1 7 dpc
++	+++	+++	+++	+++	G2 5 dpc
+++	++	++	++	+++	G2 7 dpc
+	++	+	+	++	G3 5 dpc
++	+	+	++	++	G3 7 dpc

Trachea: −, Normal tracheal mucosa and submucosa; +, Mild epithelial hyperplasia deciliation, desquamation and congestion of the mucosal blood vessels; ++, Moderate epithelial degeneration, and inflammatory cell infiltration; +++, Severe epithelial degeneration, necrosis and desquamation. Lung: -, Normal parabronchi and respiratory portions; +, Mild congestion of the interstitial blood vessels with mild parabronchial inflammatory exudate; ++, Moderate congestion and hemorrhages, collapsed and ruptured alveoli; +++, Severe hemorrhages and leukocytic infiltration. Proventriculus: -, Normal histological structure of the proventriculus; +, Mild degree of degeneration, vacuolation and mild desquamation; ++, Moderate degree of epithelial degeneration, desquamation and focal leukocytic infiltration; +++: Severe epithelial necrosis, ulcerations and desquamation with congestion and infiltration of inflammatory cells into the mucosa and submucosa of the proventriculus. Intestine: -, Normal villi and mucosal lining; +, Mild degree of enteritis (mild mucosal defects and epithelial degeneration); ++, Moderate degree of enteritis (epithelial cell denudation, inflammatory infiltration, villus fusion); +++, Severe degree of enteritis (severe necrosis, erosion, ulcers of the epithelial layer). Lymphoid follicles: -, Normal histoarchitecture of lymphoid aggregations; +, Mild degree of lymphoid depletion (mild mucosal hyperplasia, epithelial degeneration, and leukocytic infiltration); ++, Moderate degree necrosis of lymphoid follicles; +++, Severe degree of loosening or disappearance of the structure of lymphoid follicles

The tracheal tissue sections obtained from the LE-treated group at 5th dpc exhibited mild epithelium hyperplasia, with ciliated cells appearing rounded up and detached from the surface. In addition, lymphocytes had invaded the lamina propria, and an occasional germinal center was observed (Fig. 4E). Elongated and distorted mucous glands were also spotted in these tissue sections. On the other hand, at 7 dpc, the tracheal sections showed moderate epithelial deciliation, degeneration, and rounding up. Despite these changes, the epithelium remained intact, and there was only mild lymphocyte infiltrate and mild epithelial hyperplasia (Fig. 4F). The lesions scored as mild to moderate in severity at 5th and 7th dpc (Table 7).

#### Lung

The negative control group displayed normal parabronchi and patent air capillaries (Fig. 4G, H) with no pulmonary lesions noted (Table 7) at 5th and 7th dpc.

In contrast, the NDV group exhibited severe pulmonary injury (Fig. 4I) at 5th dpc with a severe score. At 7th dpc, although the severity of lesions decressed to a moderate score (Table 7), theyspread to encompass larger portions of the pulmonary tissue. Extensive bleeding was observed in the parabronchial lumen and collapsed and ruptured alveoli (Fig. 4J). Furthermore, inflammatory cells were present in the interalveolar septa at 5 dpc.

The LE-treated group showed only mild inflammatory exudate in the lumen and congested pulmonary blood vessels (Fig. 4K, L) at 7 dpc with a mild lesion score (Table 7).

#### Proventriculus

The negative control group displayed normal proventriculus epithelium, glands, and muscles at both time points, with no significant alterations observed (Fig. 4M, N).

Conversely, birds in the NDV group exhibited severe pathological changes including ulcerations, necrosis, and desquamation on epithelial surface and damage with in the glands of proventriculus at both 5 and 7dpc (Fig. 4O) with high lesion score (Table 7). Inflammatory cell infiltration into the sub mucosa of the proventriculus was also observed (Fig. 4P).

The LE-treated group exhibited moderate degeneration, vacuolation, and mild desquamation of the proventriculus glands and epithelium (Fig. 4Q), with focal congestion and inflammatory cell infiltration into the submucosa of the proventriculus at 7 dpc (Fig. 4R). However, this group also showed a slight improvement in the degenerative changes of the histological structure of the proventriculus, with only mild desquamation and congestion in the submucosa and a rare presence of inflammatory cells at 7 dpc (Fig. 4R). The lesions scored as mild (Table 7).

#### Intestine

**Fig. 5:**
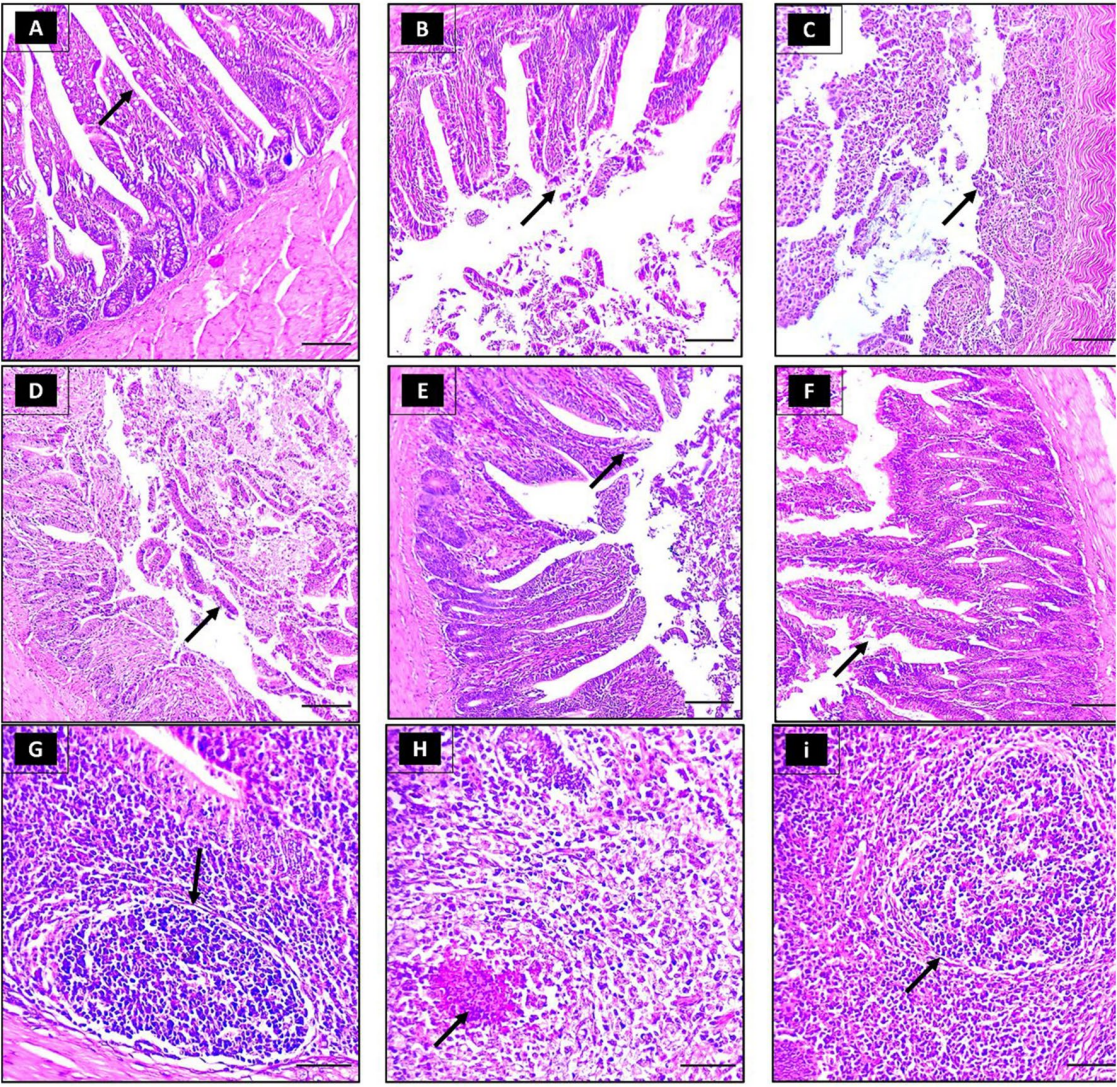
Depicts histological micrographs of different organs from chickens of distinct experimental groups (H&E [magnification, ×200]). Group 1 corresponds to the negative-control group, with specimens collected at 5- and 7-dpc displaying typical intestinal tissue with thin, finger-shaped villi lined by columnar epithelial cells with a basal nucleus (A), normal villus structures with a few denuded villi tips (B). Group 2 represents the positive-control group, with samples obtained at 5 and 7 dpc after being challenged solely with (NDV) displaying completely destroyed villi and the necrotic mucosal layer disintegrated into necrotic debris filling the intestinal lumen (C, D). Finally, group 3 is the experimental group, which received licorice in addition to NDV. Samples were collected at 5 and 7 dpc displaying intact villi with only some epithelium defects at the villus’s tip (E, F). Group 1(negative control), 2 (positive control), and 3 (NDV licorice) samples were collected at 5 and 7 dpc displaying normal (G), Depleted (H), and regenerated (i) lymphoid tissue aggregations

Intestinal sections from the control negative group at both 5 and 7 dpc showed normal tissue architecture (Fig. 5A). A few denuded villi tips were observed at 7 dpc (Fig. 5B).

In contrast, the NDV group showed destroyed villi and disintegrated necrotic mucosal layer at 5 dpc (Fig. 5C). At 7 dpc, there was necrosis of intestinal mucosa, crypt hyperplasia, and mononuclear cell infiltration (Fig. 5D).

In the LE/NDV group, villi appeared intact with only minor defects observed at the tips of some epithelial cells at 5th dpc (Fig. 5E). However, necrotic debris was observed in the lumen with mild inflammatory cell infiltration in the lamina propria. At 7 dpc, moderate focal epithelial necrosis, a fusion of villi and crypt, and inflammatory cell infiltration in the lamina propria were observed (Fig. 5F). There was moderate lesion score as less severe tissue damage and inflammation observed in the LE/NDV group compared with the NDV group with more severe lesion score (Table 7).

#### Cecal tonsils

The negative control group displayed normal histological architecture of lymphoid aggregations, indicating no significant deviations (Fig. 5G). On the other hand, the NDV group exhibited loosening or disappearance of the structure of lymphatic nodules due to severe lymphoid depletion with necrosis at both time points (Fig. 5H) and were scored as severe (Table 7). In contrast, the LE/NDV group demonstrated a reduced lymphocyte number in the lymphatic nodules at both time points, with the nodular structure remaining intact (Fig. 7I) and the lesions scored as moderate without any structural damage (Table 7).

#### Molecular docking

**Fig. 6:**
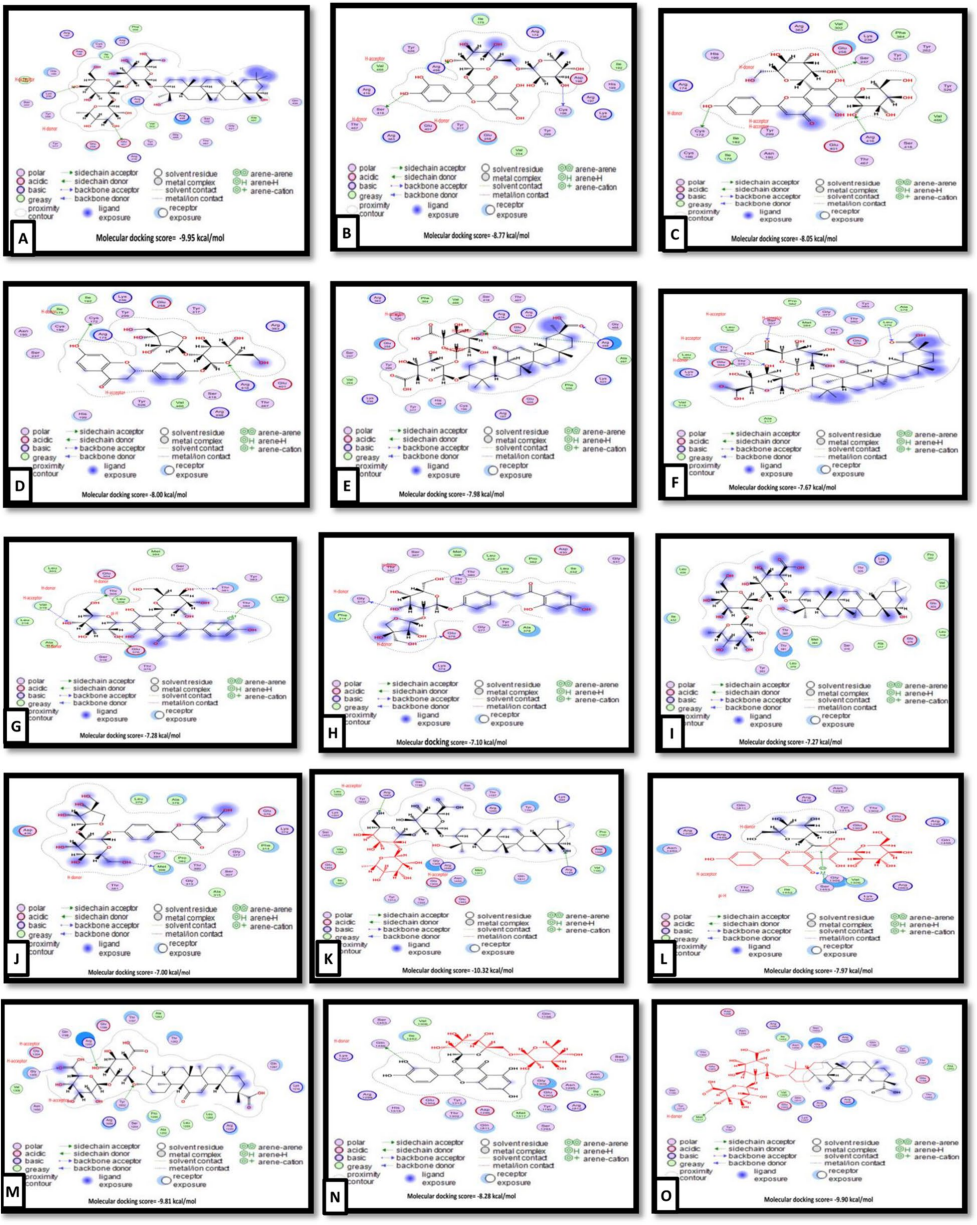
Molecular docking interaction of soyasaponin i (A), rutin (B), vicenin 2 (C), liquiritin apioside (D), and glycyrrhizin (E) with hemagglutinin-neuraminidase binding site in NDV. Molecular docking interaction of liquorice (F), vicenin 2 (G), soyasaponin i (H), licuroside (I), and liquiritin apioside (J) with fusion glycoprotein F0 binding site in NDV. Molecular docking interaction of soyasaponin i (K), glycyrrhizin (L), liquorice (M), rutin (N), and vicenin 2 (O) with fusion glycoprotein F0 binding site in NDV

**Fig. 7:**
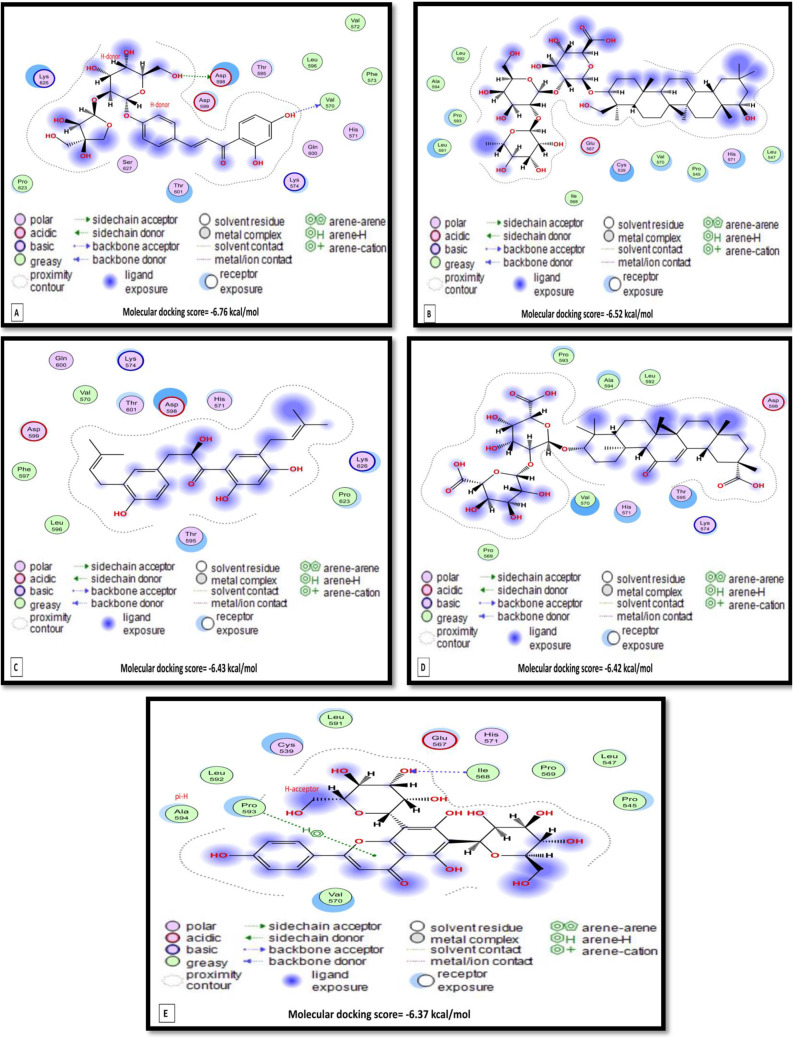
Molecular docking interaction of licuroside (A), soyasaponin i (B), kanzonol y (C), liquorice (D), and vicenin 2 (E) with chicken toll-like receptor 3 (TLR-3)

Molecular docking scores and interactions of *G. glabra* bioactive compounds against NDV’s hemagglutinin-neuraminidase, fusion glycoprotein F0, and RNA-directed RNA polymerase L and *Gallus gallus*’s toll-like receptor-3 are represented in Table 8 and the top 5 docking scores of *G. glabra* bioactive compounds (Figs. 6 and 7). Hemagglutinin-neuraminidase in NDV was targeted by soyasaponin I, rutin, vicenin 2, liquiritin apioside, and glycyrrhizin with binding energy of -9.95, -8.77, -8.05, -8.00, and − 7.98 kcal/mol, respectively (Fig. 6A-E). Liquorice, vicenin 2, soyasaponin I, licuroside, liquiritin apioside were bound to the NDV’s fusion glycoprotein F0 binding site by -7.67, -7.28, -7.27, -7.10, -7.00 kcal/mol binding energy, respectively (Fig. 6F-J). Similarly, soyasaponin i (-10.32 kcal/mol), glycyrrhizin (-9.90 kcal/mol), licorice (-9.81 kcal/mol), rutin (-8.28 kcal/mol), and vicenin 2 (-7.97 kcal/mol) have interacted with the binding site of RNA-directed RNA polymerase L (Fig. 6K-O).

**Table 8:** Molecular docking scores of *Glycyrrhiza glabra* bioactive compounds against NDV’s hemagglutinin-neuraminidase, fusion glycoprotein F0, and RNA-directed RNA polymerase L and *Gallus gallus*’s toll-like receptor-3 (TLR-3)

Lotus ID	Compounds	Molecular docking scores (kcal/mol)			
		Newcastle disease virus (NDV)			Chicken
		Hemagglutinin-neuraminidase	Fusion glycoprotein F0	RNA-directed RNA polymerase L	TLR-3
LTS0182567	**(-)-lavandulol**	-4.95	-4.57	-4.60	-4.93
LTS0072900	**(-)-naringenin**	-5.90	-5.10	-5.65	-4.60
LTS0191687	**(-)-vestitol**	-5.26	-5.08	-5.60	-4.71
LTS0158119	**3-isothujone**	-4.78	-4.51	-4.64	-5.00
LTS0135577	**3’-methoxyglabradin**	-6.28	-5.53	-6.45	-4.89
LTS0200538	**4’-o-methylglabridin**	-6.29	-5.70	-6.58	-5.19
LTS0251224	**5-deoxyflavanone**	-5.59	-4.62	-5.18	-4.77
LTS0014786	**6**,**8-diprenylgenistein**	-7.20	-6.12	-7.67	-5.90
LTS0225956	**Abyssinone ii**	-6.43	-5.32	-6.39	-5.22
LTS0275427	**Afrormosin**	-5.44	-5.01	-5.81	-5.24
LTS0044471	**Amylfuran**	-4.75	-4.65	-4.76	-4.96
LTS0068303	**Asahina**	-5.35	-4.64	-5.23	-4.87
LTS0249588	**Astragalin**	-6.66	-6.43	-6.50	-5.82
LTS0012882	**Carvacrol**	-4.96	-4.28	-4.45	-4.41
LTS0181568	**Cymene**	-4.85	-4.39	-4.41	-4.87
LTS0069837	**Cynaroside**	-6.58	-6.48	-6.88	-6.02
LTS0138668	**Echinatin**	-5.75	-5.38	-5.44	-5.12
LTS0222995	**Enoxolone**	-6.87	-6.36	-6.33	-5.12
LTS0180128	**Euchrenone a5**	-7.02	-5.73	-6.92	-5.93
LTS0048628	**Euchrestaflavanone a**	-7.66	-5.89	-7.58	-5.83
LTS0126716	**Fenchone**	-4.78	-4.53	-4.34	-3.88
LTS0082756	**Formononetin**	-5.11	-4.94	-5.51	-4.82
LTS0073369	**Formononetin 7-o-glucoside**	-6.61	-6.49	-6.72	-5.31
LTS0210648	**Galangin**	-5.11	-4.99	-5.59	-4.98
LTS0094683	**Gancaonin f**	-6.34	-5.21	-6.47	-5.44
LTS0003159	**Gancaonin g**	-6.13	-5.91	-6.36	-5.17
LTS0077774	**Gancaonin h**	-6.81	-5.99	-7.45	-5.16
LTS0072777	**Gancaonin l**	-6.18	-5.74	-6.80	-5.53
LTS0106538	**Genistein**	-5.19	-5.12	-5.49	-4.78
LTS0250433	**Glabranin**	-6.64	-5.01	-6.31	-5.37
LTS0232975	**Glabrene**	-5.84	-5.77	-6.64	-5.02
LTS0075616	**Glabridin**	-5.98	-5.48	-5.92	-5.38
LTS0151626	**Glabrocoumarin**	-6.51	-5.68	-5.93	-5.12
LTS0262018	**Glabrocoumarone a**	-6.05	-5.53	-6.14	-5.29
LTS0274460	**Glabrocoumarone b**	-6.05	-5.36	-5.94	-5.00
LTS0164961	**Glabrol**	-7.36	-5.57	-7.47	-5.86
LTS0075204	**Glabrone**	-6.37	-5.53	-6.56	-5.00
LTS0186848	**Glycycoumarin**	-6.46	-5.57	-6.92	-5.05
LTS0087818	**Glycyrin**	-7.30	-6.08	-6.89	-5.43
LTS0198644	**Glycyrrhetinic acid**	-5.83	-6.49	-6.35	-4.87
LTS0193131	**Glycyrrhisoflavanone**	-6.44	-5.74	-5.99	-4.98
LTS0121878	**Glycyrrhizin**	-7.98	-6.77	-9.90	-5.98
LTS0090907	**Glyinflanin a**	-7.37	-6.13	-6.78	-5.95
LTS0133651	**Glyinflanin b**	-6.05	-5.66	-5.87	-5.54
LTS0241667	**Glyinflanin g**	-6.69	-6.31	-6.96	-5.55
LTS0179228	**Guaiacol**	-4.49	-3.81	-4.37	-4.53
LTS0267683	**Hispaglabridin a**	-7.17	-6.03	-6.79	-5.96
LTS0155248	**Hispaglabridin b**	-7.43	-5.95	-6.60	-5.39
LTS0257369	**Hydroxywighteone**	-6.21	-6.33	-6.03	-4.90
LTS0223233	**Isobavachromene**	-6.58	-6.13	-6.31	-5.35
LTS0066952	**Isoglycycoumarin**	-6.91	-6.06	-6.60	-5.38
LTS0264727	**Isolicoflavonol**	-6.19	-6.17	-6.46	-5.95
LTS0051422	**Isoliquiritin**	-6.73	-6.69	-7.38	-5.57
LTS0254337	**Isoquercetin**	-6.70	-6.00	-6.44	-6.12
LTS0087575	**Isorhamnetin 3-galactoside**	-7.09	-6.58	-7.13	-5.51
LTS0137002	**Isorhamnetin 3-o-glucoside**	-7.30	-6.91	-7.31	-5.86
LTS0157117	**Isoschaftoside**	-7.65	-6.29	-7.92	-5.63
LTS0035187	**Isovitexin**	-6.89	-6.69	-6.61	-5.14
LTS0075703	**Kanzonol b**	-6.54	-5.69	-6.07	-5.14
LTS0266469	**Kanzonol c**	-6.84	-5.96	-7.46	-5.53
LTS0012990	**Kanzonol d**	-5.88	-5.99	-6.35	-5.54
LTS0138968	**Kanzonol y**	-7.63	-5.90	-7.14	-6.43
LTS0018267	**Kumatakenin**	-5.70	-5.59	-6.05	-5.19
LTS0106634	**Licoagrochalcone a**	-6.30	-5.92	-6.18	-5.51
LTS0020333	**Licoagrochalcone b**	-6.88	-5.99	-6.58	-5.27
LTS0187725	**Licoagrochalcone c**	-7.19	-5.85	-6.45	-5.36
LTS0270336	**Licoagrochalcone d**	-7.13	-5.97	-6.45	-5.29
LTS0018907	**Licochalcone a**	-6.23	-5.42	-6.39	-5.43
LTS0192338	**Licochalcone b**	-5.97	-5.25	-5.79	-4.78
LTS0183214	**Licochalcone c**	-6.81	-5.81	-6.92	-4.94
LTS0132019	**Licocoumarone**	-6.31	-5.20	-6.15	-4.84
LTS0244117	**Licoflavanone**	-6.37	-5.64	-6.73	-5.45
LTS0004664	**Licoflavone a**	-6.27	-6.10	-6.35	-5.03
LTS0122155	**Licoflavone b**	-6.55	-6.28	-6.92	-5.47
LTS0219719	**Licoflavonol**	-5.76	-5.77	-6.24	-5.17
LTS0263391	**Licoisoflavone a**	-6.37	-6.01	-6.40	-4.87
LTS0055944	**Licoisoflavone b**	-6.61	-5.52	-6.49	-4.90
LTS0048734	**Licopyranocoumarin**	-7.14	-5.96	-6.73	-5.17
LTS0274337	**Licoricidin**	-7.24	-6.19	-7.39	-5.86
LTS0132318	**Licuroside**	-7.85	-7.10	-7.85	-6.76
LTS0103894	**Liquiritin**	-7.14	-6.46	-6.80	-6.05
LTS0188438	**Liquiritin apioside**	-8.00	-7.00	-7.70	-5.89
LTS0142270	**Liquorice**	-7.94	-7.67	-9.81	-6.42
LTS0211446	**Lupalbigenin**	-6.93	-6.27	-7.10	-5.59
LTS0256952	**Lupeol**	-6.26	-5.52	-6.60	-4.56
LTS0229079	**Lupiwighteone**	-6.10	-5.43	-6.79	-5.21
LTS0261149	**Medicarpin**,** (-)-**	-5.40	-5.23	-5.48	-4.96
LTS0215385	**Morachalcone a**	-6.75	-5.58	-6.30	-5.14
LTS0202475	**Myrtenal**	-4.51	-4.17	-4.74	-4.02
LTS0031098	**Naringenin**	-5.49	-5.09	-6.25	-4.64
LTS0089772	**Neoliquiritin**	-6.65	-6.25	-6.89	-6.24
LTS0237730	**Odoratin**	-6.00	-5.70	-6.00	-4.81
LTS0235553	**Ononin**	-7.57	-6.79	-7.16	-5.80
LTS0014950	**Paeonol**	-5.10	-4.58	-4.82	-4.74
LTS0124936	**Parvisoflavone b**	-6.43	-5.32	-5.95	-5.44
LTS0151338	**Phaseol**	-6.17	-5.45	-6.41	-5.10
LTS0010732	**Pinit**	-4.85	-4.51	-4.75	-4.68
LTS0194724	**Pinitol**	-4.95	-4.70	-4.74	-4.64
LTS0141508	**Pinocembrine**	-4.97	-5.00	-5.48	-4.63
LTS0261766	**Prunetin**	-5.19	-5.22	-5.43	-4.58
LTS0119297	**Pseudoionone**	-5.04	-4.80	-5.51	-4.29
LTS0186298	**Quercitrin**	-6.86	-5.96	-7.18	-5.34
LTS0032845	**Rutin**	-8.77	-6.52	-8.28	-6.07
LTS0104338	**Schaftoside**	-7.23	-6.48	-7.74	-5.50
LTS0128805	**Shinflavanone**	-7.54	-6.40	-6.68	-5.26
LTS0058527	**Sophoraflavanone b**	-6.97	-5.50	-6.26	-5.19
LTS0182499	**Soyasaponin i**	-9.95	-7.27	-10.32	-6.52
LTS0152081	**Talmon**	-4.62	-4.24	-4.72	-4.40
LTS0027534	**Tephrinone**	-7.08	-5.33	-6.65	-5.68
LTS0168527	**Thymol**	-4.95	-4.41	-4.65	-4.90
LTS0267055	**Trifolin**	-7.32	-6.54	-6.75	-5.96
LTS0181160	**Vicenin 2**	-8.05	-7.28	-7.97	-6.37
LTS0136408	**Wighteone**	-5.88	-5.86	-6.85	-5.26
LTS0139725	**Xambioona**	-6.69	-6.15	-6.48	-5.33
LTS0063487	**Yinyanghuo d**	-6.00	-5.71	-6.48	-5.40

Regarding the host, licuroside, soyasaponin i, kanzonol y, licorice, and vicenin 2 interacted with the binding site of chicken TLR3 by the binding energy of -6.76, -6.52, -6.43, -6.42, and − 6.37 kcal/mol, respectively (Fig. 7).

## Discussion

*Glycyrrhiza glabra*, licorice root, is one of the world’s oldest and most well-known medicinal plants. In this study, LE contained many important secondary metabolites such as phenolics, flavonoids, tannins, saponins, and terpenoids as reported in our previous study (Elbasuni et al. 2024). Similarly water and ethanolic extracts of licorice roots were reported to contain total phenols and flavonoids near the values reported in the current work (Alfauomy et al. 2020) and (Rodino et al. 2015). Moreover, different phytochemical contents of *G. glabra* aqueous extract as total phenols, flavonoids, tannins, saponins, and terpenoids content were previously recorded (Soliman and El-Genaidy 2021). Secondary metabolites determination confirmed that LE was very rich in phenols, flavonoids, and saponin while possessing an acceptable content of tannins and terpenoids. These findings could account for many reported pharmaceutical effects of LE such as antioxidant, anticancer, antibacterial, antiprotozoal, antidiabetic, and hepatoprotective effects (Rajpurohit et al. 2017; Elbasuni et al. 2024).

The current investigation observed that subjecting SPF layer chicks to challenge with virulent genotype VII ND virus led to the development of Newcastle disease, which was confirmed by the presence of high titers of the virus in nasopharyngeal swabs, clinical symptoms, lesions, and mortality rates. It was observed that the values of these parameters were greater in the 5th dpc compared to the 7th dpc. Comparable results were documented in a prior study conducted by Omeke et al. (2018). The decline in clinical parameter values on the seventh-day post-challenge may be ascribed to the fact that layer chickens exhibit a notably elevated early HI antibody response to vvNDV infection on the 7th dpc, resulting in restricted virus replication (Omeke et al. 2018). On the other hand, intermittent aqueous medication of SPF chicks with LE (0.5 g /L) before and during the NDV challenge reduced the severity of clinical symptoms and mortality rates. Furthermore, when compared with the NDV group, LE-treated chicks had fewer macroscopical lesions on internal viscera and lower scoring values. Moreover, LE treatment had a significant and clear impact on reducing virus shedding. Besides, the molecular docking modeling revealed the binding ability of G. glabra’s active ingredients with NDV’s RNA-directed RNA polymerase L leading to lowering of their viral replication. These findings are consistent with previous study discovered that using licorice extract can inhibit the multiplication of the NDV virus in embryonated eggs (Omer et al. 2014). Furthermore, licorice extract improved cellular and humeral immunity against NDV vaccine in chickens (Wu et al. 2022). According to a large body of data accumulated in recent years, licorice and its extracted components are capable of inhibiting virus replication, direct inactivation of viruses, inhibition of viral gene replication and expression, increase in cell death, as well inhibiting inflammation (Huan et al. 2021). In addition, the molecular docking assessment in the current study revealed the ability of *G. glabra* bioactive compounds to control NDV replication and invasion.

Concerning the hemogram, chicks in NDV group displayed a substantial decline in the RBC, Hb, PCV, and MCHC values and increase in MCV that exhibits macrocytic hypochromic anemia symptoms. These results confirm those of (Adeyemo and Sani 2013; Velguth et al. 2010). The severe anemia seen in the challenged chickens could be due to intravascular hemolysis and intestinal hemorrhage (Eze et al. 2014). Also, the direct increase in the mean corpuscular volume may be correlated to the increase in reticulocyte count which could be defined as macrocytic hypo-chromic anemia since these immature RBCs are significantly larger and contain less hemoglobin content (Calderon et al. 2005).

Compared with the negative control group, the current study recorded leukocytopenia, heteropenia, lymphocytopenia, and eosinopenia in NDV-challenged birds. The leukocytic changes seen in this study came in accordance with previous work that reported presence of a significant decline in the leucogram of hens challenged with NDV (Schmidt et al. 2008). The recorded lymphocytopenia and heteropenia could be attributed to the prominent depressive and proliferative responses of chickens’ white blood cells during NDV infection (El-Mandrawy and Ismail 2017). Also, could be justified by lymphocyte depletion in the bursa of Fabricius, spleen, and thymus complex mechanisms due to endogenous corticosterone release which causes transient lymphocyte redistribution and entrapment of circulating lymphocytes within lymphoid tissue during viral infection (Ezema et al. 2009), and presence of inflammatory mediators that encourages the diapedetic movement of heterophils and lymphocytes from the blood and lymphoid tissues to the areas of inflammation where they are ultimately eliminated during NDV challenge (Ng et al. 2005).

The results of the current study showed that NDV group has significantly higher ALT, AST, glucose, uric acid, and creatinine, besides lower serum total proteins, albumin, and globulins compared with the control one. Similair results were recorded in ducks challenged with NDV (Zahid et al. 2022), and in chickens (El-Mandrawy and Ismail 2017; Eze et al. 2014). The decrease in total protein and albumin levels could be attributed to impaired liver function and protein loss due to kidney damage (Kaslow 2011); or due to insufficient take of protein in diet and diarrhea (Ihedioha and Chineme 2004). In previous investigation, greater corticosterone levels in NDV-injected birds may have contributed to the increase in glucose concentration (hyperglycemia) seen in the NDV-challenged birds (El-Mandrawy and Ismail 2017). Our findings were in line with those of Ismail (2017), who discovered hyperproteinemia and hyperglobulinemia along with increased serum uric acid and creatinine 42 days after NDV infection in broiler chickens. These attributed to the exhibited extensive intertubular blood vessel congestion, diffuse renal tubules, and glomeruli coagulative necrosis together with cystic dilatation and/or morphological deformation of some renal tubules noticed in kidney tissue of NDV-challenged birds (El-Bahrawy et al. 2016).

Lysozymes play a variety of roles, such as host defense and innate immune systems. In addition to having antibacterial properties, lysozyme also has antiviral, anti-inflammatory, anticancer, and immune-modulatory properties (Sava 1996). Our results demonstrated lower serum lysozyme activity in NDV-challenged chickens, in accordance with (El-Samadony et al. 2020), who revealed a prominent decrease in lysozyme concentration in birds challenged with the chicken anemia virus. The evidence that viral infection has an impact on the immunological profile of chicks and reports the downregulation of GM-CSF in chicks; may be the cause of the lower level of lysozyme concentration (Basaraddi et al. 2013). In comparison with the control groups, NDV challenged group showed a significantly elevated level of serum NO. Similarly, (Rehman et al. 2018) demonstrated NDV-challenged animals showed increased levels of NO in the intestinal mucosa. The viral infection stimulates NO production as it can combine with superoxide to make peroxynitrite, which upon decomposition produces more harmful free radicals and causes damage to lipid membranes, proteins, and nucleic acids be involved in the destruction of intestinal barrier function and cell death (Robinson et al. 2005).

In this study, The NDV infection demonstrated higher MDA production, along with SOD and catalase activities in the trachea, lung proventriculus, and intestine tissues, relative to that of the control. Similarly, a significant increase in the concentrations of MDA and NO along with a significant decrease in GSH concentration and activities of catalase and SOD were recorded in the brain and bursa of Fabricius of the NDV (KUDU 113)-challenged chickens at 7 dpi (Okoroafor et al. 2021). Other researchers showed an elevation of MDA levels in the brain (Subbaiah et al. 2013) and bursa (Kristeen-Teo et al. 2017) of NDV-challenged chickens. These higher generations of NO and lipid peroxide could result in higher RNS and ROS concentrations, which are crucial for NDV infection.

The immune response to a viral infection increases the production of interferon, which directly target viruses by preventing the creation of their proteins or by triggering defensive mechanisms in an indirect manner (Dziewulska et al. 2018). Similar to our results, increased IFN-γ transcription has been seen in chicken spleens and is upregulated in the spleen of hens infected with velogenic NDV strains (Rue et al. 2011). In addition, Susta et al. (2013) investigated the role of IFN-γ in NDV infection and illness development. They observed that the cytokine was expressed in large amounts in vivo, suggesting that the early increase in IFN-γ during NDV infection could just be a delayed and insufficient response to viral replication. The elevation of No level following infection was confirmed in our results.

TLRs like TLR-3 and TLR-7 are crucial for triggering the host’s innate immune system, molecular patterns linked to pathogens, such as the RNA viruses’ nucleic acids, such as NDV in mammals, domestic fowl, and insects (Kang et al. 2016). In this research, expression of the TLR-3 gene in the trachea, lung proventriculus, and intestine tissues of NDV-challenged chicken was highly upregulated compared with the control non-challenged group. These results are a line with previous study showed that Herts/33 and La Sota cell lines infected with NDV strain can boost TLR-3 expression (Cheng et al. 2014). Moreover, numerous studies have pinpointed the potential function of TLR-3 in viral defense mechanisms (Jiang et al. 2003). This is the first study to investigate the differential expression of genes in the TLR-3 and IFN-γ in the respiratory and digestive tract between NDV-infected and non-infected chickens. The results revealed that TLR-3 and IFN-γ genes were involved in response to NDV invasion in the trachea, lung, proventriculus, and intestine tissues.

LE treatment resulted in a significant decrease in serum ALT and AST activities, blood glucose level, urea, and creatinine with the significant restoration of serum proteins (total protein, albumin, and globulin) after the NDV challenge. The hepatoprotective effect of licorice is correlated to glycyrrhizic acid which promotes hepatocyte survival and stops alterations in cell membrane permeability as adding 0.4% LE to broiler chicks’ drinking water decreased ALT levels (Salary et al. 2014). In line with our findings, serum ALT, AST, and glucose concentrations were diminished in chickens treated with licorice at 0.4 and 0.8 g/L in the drinking water (Abo-Samaha et al. 2022). As well, the administration of 0.2 and 0.3 g/L of licorice extract through drinking water reduces the level of blood glucose (Moradi et al. 2013). A significant decrease in plasma urea, uric acid, and creatinine levels was also recorded in licorice-treated rats following gentamicin toxicity (Aksoy et al. 2012).

Like our results, licorice supplementation (0.4 g/L and 0.8 g/L) resulted in dose-dependent increases in the serum lysozyme activity of broiler chicks, demonstrating its immunostimulant action (Abo-Samaha et al. 2022). Additionally, licorice provides anti-arthritic action through stabilizing lysozyme enzyme activity (Mishra et al. 2011). The antioxidant power of *G. glabra* was detected in many previous findings (Dogan et al. 2018; Sen et al. 2011; Zhao et al. 2011). The results of the current investigation showed that LE possesses antioxidant capacity via a reduction in MDA and NO levels, as well as an increment in SOD and catalase activities in NDV-challenged birds. Previous study demonstrated that the addition of 7.5, and 15 g/kg of licorice root boosted the antioxidant enzyme activity and decreased the MDA level in broiler chickens (Habibi et al. 2014).

In the current work, experimental supplementation of LE was directly associated with lower expression of the gene encoding IFN-γ and TLR-3 compared with the NDV group. Similar findings reported the immunomodulatory activities of licorice extracts may also be responsible for the higher expression of IFN-γ gene in the groups infected with APMV-1 and given herbal extracts compared with the control group (Dziewulska et al. 2018). Additionally, proinflammatory cytokines like IL-1β, TNF-α, and IFN-γ were reduced in broilers fed diets enriched with *Glycyrrhiza* polysaccharide (Zhang et al. 2021), while maintaining the physiological balance, indicating that *Glycyrrhiza* effectively reduced the LPS-induced inflammatory response. A previous investigation also showed that the use of *Glycyrrhiza* extract in the broiler diet has been found to dose-dependently down-regulate the expression of TLR-4 (Ibrahim et al. 2020). Even after *C. jejuni* infection, LE supplementation significantly reduced TLR-4 and pro-inflammatory cytokines (IL-1), indicating that it had an anti-inflammatory impact.

The histopathological examination of the respiratory and digestive tissue sections from negative control, NDV, and LE/NDV groups shed light on the pathophysiology and therapeutic potential of the LE against NDV challenge in chickens. The recorded histopathological changes in trachea and lungs of NDV group at 5th and 7th dpc were in harmony with those of (Etriwati Ratih et al. 2017). The pathological findings in trachea of NDV challenged chicks could be attributed to the tendency of the host body to shield the epithelial surface against virus invasion and attachment by inducing congestion and heightened secretion of mucous exudate (Kaspers et al. 2021). The severe pulmonary injury at 5 dpc, which progressed to affect larger areas of pulmonary tissue at 7 dpc in NDV group resulted from circulatory disturbance that caused by viremia and secondary bacterial infection (Lopez 2012). At 7th dpc, the LE/NDV group exhibited mild to moderate alterations in tracheal and pulmonary tissues. The protective effect of *G. glabra* on pulmonary tissue has been previously documented (Liu et al. 2019b). Specifically glycyrrhizin, a bioactive component of *G. glabra*, which has been to attenuate local fibrosis and pulmonary edema induced by bleomycin. Moreover, it significantly reduces the levels of collagen I and hydroxyproline in lung tissue (Gao et al. 2015).

Regarding to the digestive tract, NDV group displayed severe pathological changes in the proventriculus, including ulcerations, necrosis, desquamation of proventriculus glands and epithelium, and inflammatory cell infiltration into the submucosa in alignment with a previous report (Kabiraj et al. 2020). Also, the intestine showed rigorously destroyed villi, disintegrated necrotic mucosal layers, necrosis of intestinal mucosa, crypt hyperplasia, and mononuclear cell infiltration, as previously reported (Rehman et al. 2018). In contrast, the LE/NDV group exhibited mitigated histopathological alterations compared to NDV group. Specifically, there was a notable reduction in the severity of degeneration, vacuolation, and epithelial desquamation within the proventriculus glands and epithelium. The limited presence of inflammatory cells within the proventricular submucosa suggests a potential therapeutic efficacy of LE in attenuating inflammatory responses. Furthermore, the LE treatment resulted in subtle histopathological changes in intestinal sections, characterized by preserved intestinal tissue architecture and a concomitatnt decrease in inflammation. These observations are likely attributable to documented anti-inflammatory properties of *G. glabra*, including its ability to modulate CD4 + T-cell function and mitigate tumor necrosis factor-mediated cytotoxicity, as evidenced by previous investigations (Yoshikawa et al. 1997). Moreover, *G. glabra* has been reported to possess immunomodulatory and antioxidant activities (Alagawany et al. 2019), as well as protective effects on the intestinal barrier function (Shi et al. 2022). Collectively, these pharmacological attributes of *G. glabra* may contribute to its capacity to mitigate the development of severe histopathological lesions.

Concerning to the histopathological examination of the lymphoid tissue, lymphocyte depletion observed in the challenged groups is a typical hallmark of virulent NDV strains, according to previous studies (Brown et al. 1999). However, treatment with LE demonstrated a significant reduction in pathological changes in the cecal tonsil lymphoid tissue. It effectively protected the tissue from structural damage and decreased lymphocyte depletion to a mild extent. This protective effect of LE treatment on cecal tonsils may be attributed to the known antiviral and immunostimulatory properties of Glycyrrhizin, as reported in both in vitro and in vivo studies. These properties enable LE treatment to inhibit the cytopathic effect, enhance antibody production in vaccinated groups, and stimulate a notable increase in lymphocyte proliferation (Ocampo et al. 2016; Soufy et al. 2012).

## Conclusion

In response to the worldwide demand for organic poultry products that are safe for consumers and affordable for poultry producers, scientists have spent decades scouring nature for natural plants that deliver treatment effects for various infectious diseases afflicting the poultry industry. Intermittent administration of 0.5 g of LE / L drinking water to SPF chicks before the NDV challenge and after the onset of the challenge for 5 days resulted in very positive effects in terms of reducing the clinical picture of the disease, virus shedding, clinicopathological damages, and IFN- and TLR-3 gene expression. In addition, LE mitigated the macroscopic and microscopic harm to the respiratory, digestive, and lymphoid organs. Also, the molecular docking study indicated the efficacy of *G. glabra* to interact with NDV target proteins and chicken TLR-3 hindering NDV replication and spread. We highly recommend conducting additional field research on the product using commercial chickens to evaluate its effectiveness in reducing the deleterious effects of Newcastle virus infection.

## Data Availability

No datasets were generated or analysed during the current study.
